# Attempted Transmission of Marburg Virus by Bat-Associated Fleas *Thaumapsylla breviceps breviceps* (Ischnopsyllidae: Thaumapsyllinae) to the Egyptian Rousette Bat (*Rousettus aegyptiacus*)

**DOI:** 10.3390/v16081197

**Published:** 2024-07-25

**Authors:** Janusz T. Pawęska, Nadia Storm, Petrus Jansen van Vuren, Wanda Markotter, Alan Kemp

**Affiliations:** 1 Centre for Emerging Zoonotic and Parasitic Diseases, National Institute for Communicable Diseases of the National Health Laboratory Service, Sandringham 2131, South Africa; nstorm@bu.edu (N.S.); petrus.jansenvanvuren@csiro.au (P.J.v.V.); alank@nicd.ac.za (A.K.); 2 Centre for Viral Zoonoses, Department of Medical Virology, Faculty of Health Sciences, University of Pretoria, Pretoria 0001, South Africa; wanda.markotter@up.ac.za; 3 Faculty of Health Sciences, University of Witwatersrand, Johannesburg 2050, South Africa; 4 Department of Microbiology, School of Medicine, Boston University, Boston, MA 02118, USA; 5 CSIRO Health & Biosecurity, Australian Centre for Disease Preparedness, Geelong, VIC 3220, Australia

**Keywords:** Marburg virus, bat fleas, Egyptian rousette bat, transmission, vectorial capacity

## Abstract

Egyptian rousette bats (ERBs) are implicated as reservoir hosts for Marburg virus (MARV), but natural mechanisms involved in maintenance of MARV in ERB populations remain undefined. A number of hematophagous ectoparasites, including fleas, parasitize bats. Subcutaneous (SC) inoculation of ERBs with MARV consistently results in viremia, suggesting that infectious MARV could be ingested by blood-sucking ectoparasites during feeding. In our study, MARV RNA was detected in fleas that took a blood meal during feeding on viremic bats on days 3, 7, and 11 after SC inoculation. Virus concentration in individual ectoparasites was consistent with detectable levels of viremia in the blood of infected host bats. There was neither seroconversion nor viremia in control bats kept in close contact with MARV-infected bats infested with fleas for up to 40 days post-exposure. In fleas inoculated intracoelomically, MARV was detected up to 14 days after intracoelomic (IC) inoculation, but the virus concentration was lower than that delivered in the inoculum. All bats that had been infested with inoculated, viremic fleas remained virologically and serologically negative up to 38 days after infestation. Of 493 fleas collected from a wild ERB colony in Matlapitsi Cave, South Africa, where the enzootic transmission of MARV occurs, all tested negative for MARV RNA. While our findings seem to demonstrate that bat fleas lack vectorial capacity to transmit MARV biologically, their role in mechanical transmission should not be discounted. Regular blood-feeds, intra- and interhost mobility, direct feeding on blood vessels resulting in venous damage, and roosting behaviour of ERBs provide a potential physical bridge for MARV dissemination in densely populated cave-dwelling bats by fleas. The virus transfer might take place through inoculation of skin, mucosal membranes, and wounds when contaminated fleas are squashed during auto- and allogrooming, eating, biting, or fighting.

## 1. Introduction

The roosting habitats of Egyptian rousette bats (ERBs; *Rousettus aegyptiacus*) have been epidemiologically associated with transmission of Marburg virus (MARV) to humans [1,2,3,4,5,6,7,8], and ecological and experimental studies implicate ERBs as reservoir hosts for marburgviruses [9,10,11,12,13,14,15,16,17,18,19,20,21,22]. However, the exact mechanisms involved remain elusive. Subcutaneous inoculation of ERBs with MARV results in moderate viremia, replication of the virus in different tissues, and oral, faecal, and urine shedding [17,18,19,20,21,22]. Oral and faecal shedding of MARV has been demonstrated in both wild-caught and experimentally infected captive-bred ERBs [5,16,18,19,20,22]. Amman et al. [22] demonstrated that MARV-infected ERBs can shed virus onto discarded fruits and that infectious MARV is stable for 6 h or longer on tropical fruits. These findings suggest that the consumption or handling of fruit that had been test-bitten or discarded by ERBs is a risk factor for MARV spillover to humans and wildlife. An ERB cave roost consists of closely packed bats, conducive to transmission of marburgviruses via the faecal–oral route; direct contamination of open wounds from infectious saliva, faeces, or urine; and exposure to infective fomites. However, experimental studies that attempted horizontal transmission of MARV between infected and naïve in-contact ERBs have yielded conflicting results [17,19,20]. Moreover, viral shedding patterns described in experimentally infected ERBs do not necessarily reflect those occurring in natural settings. Thus, it remains questionable whether transmission by bodily fluids constitutes a plausible mechanism for sustained enzootic circulation of MARV.

MARV has been shown to infect the reproductive tracts of experimentally infected male and female ERBs [17] and both MARV and Ebola virus (EBOV) have been isolated from human seminal fluids months after clinical recovery [23,24]. Furthermore, sexual transmission has been documented for EBOV and, in the case of the 2014–2016 West African Ebola virus disease (EVD) outbreaks, was associated with transmission from clinically well male survivors [25,26,27,28,29,30,31]. Riesle-Sbarbaro et al. [32] demonstrated that experimental infection of Angolan free-tailed bats (*Mops condylurus*) with EBOV resulted in disseminated viral replication and virus shedding from both oral and rectal mucosae, without clinical disease. Moreover, they recorded specific tissue tropism for the placenta and the ability of EBOV to traverse the placenta and infect and persist in foetal tissues of *M. condylurus*. These findings are the first to demonstrate plausible routes of horizontal and vertical transmission of a filovirus in bats and suggest a role for maintenance of the filovirus in a wild reservoir host. It the context of these findings, which corroborate and confirm results of earlier experimental studies by Swanepoel et al. [33], it is worth mentioning that circumstantial evidence has implicated *M. condylurus* as the source of the 2013–2016 EVD outbreaks [34].

Bats are hosts to a number of ectoparasites, including ticks, mites, flies, and fleas [35,36,37,38] which potentially might have ecological and public health significance as biological or mechanical vectors of zoonotic agents. Although the importance of identifying ectoparasite distribution, diversity, and host–parasite associations has been recognized, vector–host–pathogen interactions among bats and their parasites are largely understudied [39]. At present, limited knowledge exists regarding the role of bats as reservoirs for arthropod-borne pathogens, which represent a substantial proportion of zoonoses worldwide [40,41]. The role of ectoparasites in filovirus transmission has been postulated [42], but only limited experimental work has been carried out to date [43], partially due to the difficulties and limitations in performing vector competence studies under biosafety level four (BSL-4) requirements and conditions.

Ecological, experimental, and discovery research point to bats as the natural reservoir hosts for filoviruses [44,45,46,47,48], but the potential for bat-associated arthropods to act as reservoirs or intermediate hosts is uncertain. Kunz and Hofmann [49] reported successful replication of MARV in experimentally inoculated *Aedes aegypti* but not in *Anopheles maculipennis* and *Ixodes ricinus,* yet tests of various arthropod species collected during filovirus outbreaks in southern Africa [50], Kenya [51], Democratic Republic of the Congo (DRC) [52,53], and Cameroon [53] have only yielded negative results. Limited numbers of nycteribiid flies and larger collections of argasid ticks from ERBs and cave walls at the Python Cave roost in Uganda tested negative for marburgviruses [5,11,54]. Of 1693 nycteribiid bat flies (*Eucampsipoda africana)* collected from ERBs roosting at Matlapitsi Cave in South Africa, where enzootic MARV circulation in ERBs has been demonstrated [14,15], 0.3% tested positive for the presence of MARV RNA [55]. However, results of vector competence studies using *E. africana* seem to indicate that these flies instead act as mechanical rather than biological vectors. Nevertheless, their ecological significance in the enzootic maintenance of MARV should not be underestimated [55]. Isolation of novel viruses such as orthoreovirus [56], orthobunyavirus [57], and adenovirus [58] from *E. africana* collected from ERBs at Matlapitsi Cave demonstrate that bat-associated nycteribiid flies may play a role as biological vectors of bat-borne arthropod viruses. Lloviu virus (LLOV) RNA was detected in *Nycteribia schmidlii* and *Ixodes simplex* collected from PCR-positive *Miniopterus schreibersii* bats in Hungary, suggesting that bat-associated parasites might play a role in the ecology of filoviruses in temperate climatic regions [59].

The colony of ERBs roosting at Matlapitsi Cave is infested by several ectoparasites throughout the year, including fleas [55]. Fleas (Order Siphonaptera) are some of the most common obligate parasites of small mammals and are important vectors of zoonotic diseases. The past decades have seen a dramatic change in the geographic and host ranges of many vector-borne pathogens, and incidences of flea-borne diseases are anticipated to rise worldwide as a result, mainly caused by the effects of climate change and the anthropogenic destruction of natural wild habitats [60,61]. Novel species, novel ectoparasite–bat-pathogen associations, and geographic changes in flea distributions, ranging from broad, across-biome distributions to narrower distributions within one or two biomes [62,63,64,65,66,67,68,69,70,71,72,73,74,75,76,77,78], are recorded globally.

Fleas are known to carry a variety of zoonotic bacterial pathogens, including *Yersinia pestis* [79,80]), *Rickettsia,* and *Bartonella* [81,82,83,84]. While fleas are implicated in the transmission of viral pathogens, including tick-borne encephalitis virus [85], feline leukemia virus [86], feline calicivirus [87], canine distemper virus [88], and myxoma virus [89], in general little is known about the diversity and abundance of flea-borne viruses. A meta-transcriptomics study on arthropod-associated viruses in fleas indicates that they can harbor a wide range of viruses [90]. A study by Villar et al. [91] implicated the domestic cat flea, *Ctenocephalides felis,* as potential vector of SARS-CoV.

Fleas are rarely host specific, but some species prefer a particular host group [92], as is the case for flea species in the family Ischnopsyllidae Wahlgren, 1907, comprised of bat-specific fleas. *Thaumapsylla breviceps breviceps* Rothschild 1907 (Ischnopsyllidae; Thaumapsyllinae) is primarily parasitic on pteropodid bats [78,93,94,95,96].

Irrespective of the geographic and historic origin of the MARV isolate used and the experimental conditions, subcutaneously inoculated ERBs invariably develop a moderate viremia [17,18,19,20]. These findings imply that blood-sucking ectoparasites might be involved in transmitting MARV in nature. Accordingly, our study aimed to determine the vector competence of *T. breviceps* for replication and transmission of MARV after feeding on MARV-viremic ERBs and in fleas that are infected with the virus through intracoelomic inoculation.

## 2. Materials and Methods

### 2.1. Source of Bats and Fleas

ERBs and bat fleas were collected at Matlapitsi Cave, located in the rural Matlapitsi Valley (24°1′ S, 30°10′ E) in the Limpopo province of South Africa. The physical and environmental characteristics of this cave have been described previously [55]. At the time of collection of bat fleas for vector competence studies, the annual average temperature recorded at the roost of the cave was 21.8 °C with a minimum of 18.5 °C and maximum of 34.6 °C. The annual average relative humidity (RH) was 86.8% with minimum of 48.4% and maximum of 98.6% [55]. Infestation of bats by different ectoparasites, including bat fleas, is noted throughout the year at Matlapitsi Cave [55], and despite rather heavy parasitemia rates, the sampled bats appeared clinically healthy. Fleas collected from the ERBs were preserved in 100% ethanol, cleared in cedar wood oil, and mounted in Canada balsam. Bat fleas were identified using the Segerman taxonomic key [97].

### 2.2. Collection of Bat Fleas from Egyptian Rousette Bats

Trapping of bats, collection of fleas from ERBs captured at Matlapitsi Cave, transportation to the BSL4 at the National Institute for Communicable Diseases of the National Health Laboratory Service (NICD-NHLS) in Johannesburg, experimental processing and testing, was done as previously described [55].

### 2.3. Infestation Rate of Egyptian Rousette Bats by Bat Fleas

The bat flea infestation rate of ERBs roosting at Matlapitsi Cave was calculated based on collections made from 11 October to 14 November 2016 using trapping and sampling procedures as previously described [55].

### 2.4. Marburg Virus Infection Rate in Bat Fleas

A total of 493 bat fleas collected from ERBs roosting at Matlapitsi Cave between June 2012 and July 2018 was processed for detection of MARV RNA as previously described [17,55].

### 2.5. Experimental Animals

The source of ERBs and trapping procedures were the same as described previously for the experimental infection study with MARV [17,19]. Wild-caught bats were transported to a BSL3 animal quarantine facility, where they were tested to prove viro- and seronegativity and then moved to a flight cage as previously described [17,19]. After quarantine and testing, MARV-seronegative bats were transported from the flight cage to the BSL4 laboratory, where they were acclimatized to the environment for one week before the experimental procedures started.

All housing, husbandry procedures, environmental conditions, and monitoring of the development of clinical signs, as well as food intake, were identical to those previously described [55]. During the time of experimentation, the average RH in the two animal isolators housing the experimental flea-infested bats in cages was 41.6 ± 16.3 SD (mean ± standard deviation) and the average temperature was 20.2 ± 0.95 °C and 21.1 ± 2 °C, respectively. The average measurements of humidity and ambient temperatures in animal isolators were within the range recorded in Matlapitsi Cave.

### 2.6. Challenge Virus

A low passage (#2 in Vero cells) of the SPU 148/99/1 isolate of MARV isolated from the serum of a patient who contracted a fatal MVD disease in 1999 in Watsa, DRC, was used for subcutaneous (SC) inoculation of bats and for intracoelomic (IC) inoculation of bat fleas as described before for bat flies [55].

### 2.7. Experimental Infections

**Figure 1:**
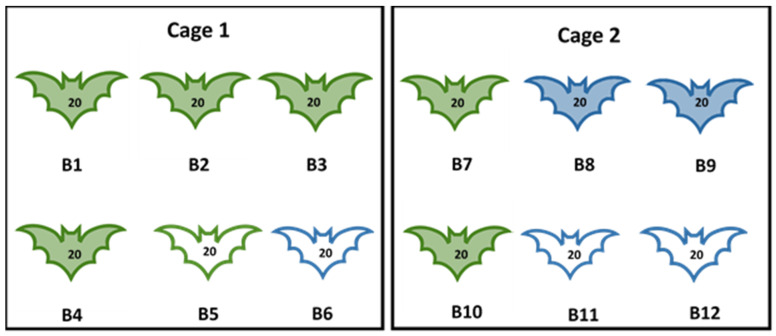
Experiment I. Number, sex of bats, MARV inoculation status, and the number of bat fleas released on Egyptian rousette bats. B = bat identification number 1–12; green outline = female; blue outline = male; number on the bat = number of fleas released on each bat; coloured-in bats = bats inoculated with Marburg virus; uncoloured bats = mock-inoculated bats.

Experiment I: This experiment aimed to assess the oral susceptibility of bat fleas to MARV following feeding on viremic bats and to attempt transmission of the virus by bat fleas to in-contact MARV-negative control bats. A total of 12 MARV-naïve ERBs, aged 8–12 months, were used in this experiment. Bats were equally subdivided into two groups and housed in cage 1 (C1) and cage 2 (C2). Each group of bats was artificially infested with a total of 240 bat fleas (20 per bat) from Matlapitsi Cave as described in Section 2.2. The following day, bat numbers B1-B4 in C1 and bat numbers B7-B10 in C2 were inoculated subcutaneously with 100 µL of tissue culture supernatant containing 10^5.3^ mL TCID_50_ of MARV, and the remaining 4 ERBs (bats B5, B6, B11, and B12) were mock-inoculated with 100 µL of Eagles Minimal Essential Medium (EMEM). The sex of experimental animals, their MARV-inoculation status, and the number of fleas released on each bat in each cage is given in Figure 1.

**Figure 2:**
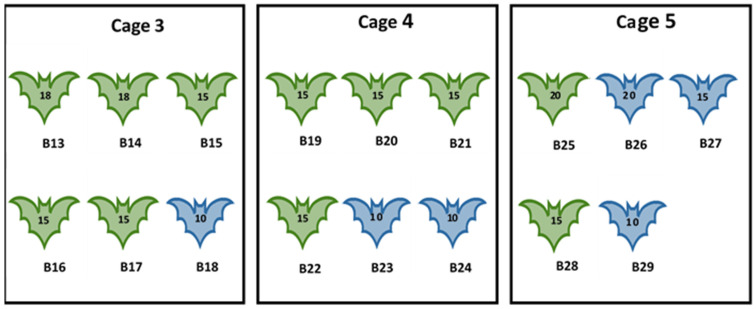
Experiment II. Number, sex of bats, and the number of MARV-inoculated fleas released on Egyptian rousette bats. B = bat identification number 13–29; green outline = female; blue outline = male; number on the bat = number of MARV-inoculated flies released on each bat.

Experiment II: This experiment aimed to investigate susceptibility of bat fleas to MARV following intracoelomic (IC) inoculation and to attempt transmission of the virus from intracoelomically inoculated fleas to MARV-naïve bats. A total of 17 ERBs, aged 8–12 months, were used in this experiment. Bats were subdivided into three groups and housed in cages C3 (n = 6), C4 (n = 6), and C5 (n = 5). All bats were first artificially infested with fleas collected from Matlapitsi Cave as described in Section 2.2. A total of 251 bat fleas (10 to 20 fleas per bat) were released on bats housed in C3–C5. All fleas were then recaptured 2–3 days after initial infestation and inoculated intracoelomically with MARV. CO_2_ anaesthesia and inoculation of fleas followed the same procedure as described previously for bat flies [55]. Fleas were inoculated intracoelomically with 0.34 ± 0.02 μL inoculum containing 10^5.3^ TCID_50_/mL of MARV (10^1.83±0.64^ TCID_50/_fly). Following inoculation, fleas were placed in individual tubes for recovery before being released onto bats. Sex and numbers of experimental ERBs in each cage, and the number of intracoelomically MARV-inoculated fleas released on bats are given in Figure 2.

Anaesthetic procedure prior to inoculation and specimen collection, sampling of bats for serological and molecular testing, and termination of the experiments were performed as previously described [17,19]. Experiment I was terminated 40 days after SC inoculation and experiment II 38 days after IC inoculation. All animals were monitored for viremia, seroconversion, food uptake, behavioural changes, and clinical symptoms.

### 2.8. Serology

An indirect enzyme-linked immunosorbent assay (I-ELISA) based on purified recombinant MARV glycoprotein antigen (recGP I-ELISA) (Integrated BioTherapeutics, Gaithersburg, MD, USA) was used for the detection of anti-MARV IgG antibody in bat sera using a previously described procedure [19].

### 2.9. Real-Time Quantitative Reverse-Transcription Polymerase Chain Reaction

Real-time quantitative reverse-transcription polymerase chain reaction (qRT-PCR) was used for the detection of MARV RNA in fleas as described previously [17]. Samples with cycle threshold values ≤ 40 were regarded as positive. RNA copy numbers detected in samples were converted into median tissue culture infectious dose (TCID_50_) genome equivalent [19].

## 3. Results

### 3.1. Identification and Morphometric Measurements of Bat Fleas

**Figure 3:**
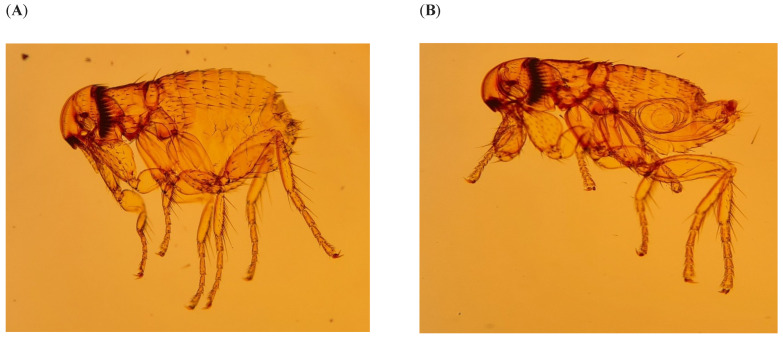
Female (**A**) and male (**B**) bat fleas *Thaumapsylla breviceps breviceps* collected from *Rousettus aegyptiacus* bats at Matlapitsi Cave, Matlapitsi Valley, Limpopo Province, South Africa.

Of 15 female and 26 male fleas (Table 1) collected from ERBs at the Matlapitsi Cave, all were identified as *Thaumapsylla breviceps breviceps* (Figure 3). Morphometric measurements of the fleas are given in Table 1.

### 3.2. Estimation of Infestation Rate of Egyptian Rousette Bats by Bat Fleas

Estimates of infestation rates and the number of bat fleas found on the ERBs in Matlapitsi Cave were calculated based on collections made from 11 October to 14 November 2016. Irrespectively of sex, the infestation rate by *T. breviceps* was very high, ranging from 95.8% (males) to 97.6% (females). The overall abundance of fleas per bat was 4.46 ± 3.52 and was comparable between male (4.43 ± 3.27) and female (4.47 ± 3.63) bats (Table 2). All infested bats appeared healthy.

### 3.3. Field Infection Rate with Marburg Virus in Bat Fleas

Of a total of 493 bat fleas collected from ERBs roosting at Matlapitsi Cave from June 2014 to July 2018, all tested negative for the presence of MARV RNA.

### 3.4. Survival of All Fleas

Of the 653 field-collected bat fleas used for the vector competence study, 93 (14.2%) died within approximately 9–10 h, between the time of collection and the time of their release on bats in the BSL4 laboratory. Of a total of 320 fleas released on the bats in cages C3–C5, 282 (88.1%) could be recaptured alive for inoculation 2–3 days after initial infestation. Of a total of 282 intracoelomically inoculated fleas, 15 were immediately stored at −70 °C until testing. Bat fleas that died after IC inoculation (n = 16) were discarded.

### 3.5. Immune Responses and Viremia in MARV-Inoculated and Control Bats

All bats remained clinically well and maintained normal water and food uptake. The kinetics of immune responses and viremia in subcutaneously inoculated bats were similar to those reported in previous experiments [17,19,55]. By day 12, all the subcutaneously inoculated bats in cages C1 and C2 became MARV-seropositive with the highest levels of anti-MARV IgG levels recorded 2–3 weeks post-inoculation. All bats in C1 and C2 became viremic within 3–5 days post-inoculation (PI), with the highest mean viremia of 10^4.06± 0.61^ TCID_50_/mL recorded on day 5 PI. Most bats cleared detectable viremia by day 12 after SC inoculation (Table 3A). Neither viremia nor seroconversion was detected in control bats.

### 3.6. Virological Findings in Fleas Fed on MARV-Viremic Bats

Blood meal sizes taken by fleas during feeding on bats are unknown and could not be determined in this study. Hinkle et al. [98] demonstrated that an average daily blood ingestion by cat fleas fed on cats was 6.97 µL of blood per flea. According to the biometrical data of fleas from different geographic populations, adult cat fleas measure 1.4 to 3 mm (mm) in length and 0.6 to 1.4 mm in width. The average length of female cat fleas is 2.35 mm and that of males is 1.77 mm [99]. In comparison to the average size of adult cat fleas, bat fleas collected at Matlapitsi Cave are about 2.5 times smaller (Table 1). When extrapolating the above-mentioned data on the blood meals [98] and body sizes of cat fleas [99], a bat flea *T. breviceps* would consume daily ~2.8 µL of blood. We used the estimated blood meal size of *T. breviceps* for the interpretation of our virological results in bat fleas fed on viremic bats.

The mean level of viremia in subcutaneously inoculated bats ranged from 10^1.13^ on day 12 to 10^4.06±0.61^ log_10_TCID_50_/mL blood on day 5 after SC inoculation (Table 3A). The mean viral load in bat fleas fed on viremic bats ranged from 10^0.6±0.37^ on day 5 to 10^1.71±0.87^ on day 7 TCID_50_/fly after SC inoculation (Table 3B). Fleas with the highest recorded viral loads ≥ 10^2.5^ would have to ingest ≥3 µL of viremic blood having ≥10^5.0^ TCID_50_/mL of the virus. Therefore, our results indicate that even the highest viral loads recorded in fleas represent the amount of the virus taken from viremic bats in the estimated size of the blood meal, rather than provide evidence for MARV replication. If fleas could support MARV replication, one would expect that, at least in some percentage of fleas fed on viremic bats, the MARV concentration would be higher than that estimated from the amount of blood taken at the peak of viremia and would remain MARV-positive through the duration of the experiment. However, only 5% of fleas tested positive for MARV on day 11 PI, and all were negative for the presence of the virus on days 14–25 PI (Table 3B). Fleas feed daily on their hosts, and the higher concentration of MARV in fleas recorded on day 7 PI might result from an accumulation of the virus from the previously taken infectious blood meals. Even though some level of transient MARV replication would have occurred in the cells of the midgut, it did not result in disseminated infection and establishment of lasting infection (Table 3B). Moreover, the lack of susceptibility to oral infection with MARV upon feeding on viremic bats and the lack of vectorial capacity of fleas to horizontally transmit MARV is further substantiated by the fact that none of naïve in-contact bats developed viremia or seroconverted within 40 days despite active movement of fleas between infected and control bats.

### 3.7. Virological Findings in Fleas Inoculated with MARV

All fleas tested on day 0 of IC inoculation were positive for MARV RNA with a mean of 10^1.67±0.42^ log_10_TCID_50_/flea, which demonstrated successful artificial delivery of the virus. Fleas tested positive for MARV RNA on day 7 (8.3%), 10 (9.6%) and 14 (3.3%) after IC inoculation with viral load decreasing from 10^1.38±0.85^ (day 7 after IC inoculation) to 10^0.54^ (day 14 after IC inoculation) log_10_TCID_50_/flea, and all were negative on days 21–24 after IC inoculation (Table 3). Viral loads recorded in fleas on day 0 of IC inoculation were consistent with, and those recorded on days 7–14 PI were lower than, the dose administered in the inoculum. None of the 17 MARV-naïve bats became viremic or seroconverted within 38 days after infestation with MARV-inoculated fleas.

**Table 3:** Quantitative reverse-transcription PCR (RT-PCR) results in the blood of captive-bred *Rousettus aegyptiacus* inoculated subcutaneously with Marburg virus (MARV) (A), in bat fleas *T. breviceps* fed on MARV-inoculated bats (B), and in bat fleas inoculated by intracoelomic administration of MARV (C).

(A)		
Days post-inoculation ^a^	Number of bats tested/positive (% viremic bats)	Mean log_10_TCID_50_^b^ ± SD ^c^/mL blood
3	6/6 (100)	3.25 ± 0.63
5	6/6 (100)	4.06 ± 0.61
7	3/6 (50)	2.05 ± 0.57
9	1/6 (16.7)	1.76
12	1/12 (8.3)	1.13
15–21	0/12	
(B)		
Days post-inoculation ^d^	Number of fleas tested/positive (% positive)	Mean log_10_TCID_50_ ± SD/flea
3	10/0	
5	14/4 (28.6)	0.6 ± 0.37
7	18/6 (33.3)	1.71 ± 0.87
11	20/1 (5)	0.64 ± 0.37
14	20/0	
21	21/0	
25	17/0	
(C)		
Days post-inoculation ^e^	Number of fleas tested/positive (% positive)	Mean log_10_TCID_50_ ± SD/flea
0	15 ^f^/15 (100)	1.67 ± 0.42
7	24/2 (8.3)	1.38 ± 0.85
10	31/3 (9.6)	1.07 ± 0.66
14	30/1 (3.3)	0.54
21	21/0	
24	10/0	

^a^ Cumulative qRT-PCR results (Experiment I—bat cages 1 and 2) in 12 *Rousettus aegyptiacus* bats inoculated subcutaneously with Marburg virus (MARV) on different days post-infection; ^b^ MARV RNA copy numbers detected in samples converted into median tissue culture infectious dose (TCID_50_) genome equivalents [17]; ^c^ standard deviation; ^d^ cumulative qRT-PCR results (Experiment I—bat cages 1 and 2) in bat fleas collected from *R. aegyptiacus* bats on different days post-infection; ^e^ cumulative MARV qRT-PCR results (Experiment II -bat cages 3, 4 and 5) in bat fleas collected from MARV-seronegative bats artificially infested with MARV-inoculated fleas; ^f^ each flea was administered 10^1.83±0.6^ TCID_50_ of MARV.

## 4. Discussion

In recent years, new and genetically highly divergent filoviruses have been reported from different continents and species, including not only bats [100,101,102,103,104,105,106,107,108] but also fishes [109,110,111,112] and a reptile [113]. For all known orthoebolaviruses (Bombali, Bundibugyo, Ebola, Reston, Sudan, Taï Forest viruses) [47], there is no convincing evidence identifying a natural reservoir host, nor the mechanisms of virus circulation in nature. Based on currently available serological and molecular findings in fruit and insectivorous bats, it appears that there might be no main or sole reservoir host for specific filovirus species but rather a complex multi-taxonomic ecosystem supporting their perpetuation. Some researchers even argue that bats themselves might not be the ultimate reservoir hosts [114,115,116,117].

Results from experimental studies confirm that clinical, virological, and immunological responses are coherent with requirements for ERBs to be regarded as plausible natural reservoir hosts for MARV [16,17,18,19,20,21]. A remarkably relevant finding is the high reservoir competence that ERBs have demonstrated exclusively to MARV experimental infection but not for other filoviruses [118,119]. Similar results were demonstrated in *M. condylurus* for EBOV in which the virus replicated to high levels in various tissues with shedding in saliva, urine, and faeces but without clinical disease, whereas MARV, Taï Forest, and Reston viruses failed to establish productive infections in this bat species [32].

SC inoculation of ERBs with MARV results in viremia and replication of the virus in multiple tissues, including organs compatible with viral shedding (salivary gland, kidney, bladder, intestines, reproductive tract) [11,17,19,20,21,22]. These findings suggest that both horizontal and vertical transmission mechanisms for MARV maintenance in the ERB population might play a role. Horizonal transmission (via infectious secretions and excretions) resulting in persistent infection of immune privilege sites or the cellular immune system acting as latent reservoirs for vertical (sexual) transmission is not novel for RNA viruses, and it is well documented for both human and animal RNA viruses [120,121,122,123,124,125,126,127,128,129].

In a study by Paweska et al. [19], neither seroconversion nor viremia was demonstrated in in-contact naïve ERBs up to 42 days after exposure to MARV subcutaneously infected bats with detectable viral shedding in saliva, faeces, and urine in donor bats, thus potentially exposing control bats to infectious secretions and excretions and contaminated fomites in confined cages. Results of an experimental study by Schuh et al. [20] suggest the occurrence of a prolonged incubation period (up to 7–8 months) in bats that are naturally infected with MARV, but the mechanism remains unclear. The establishment of persistent infection in immune-privileged sites appears to be one of the plausible explanations; the latent presence of filoviruses in human tissues, e.g., in testes, has been reported [130]. However, a long-lasting incubation of MARV does not seem to be the case for ERBs that were infected under natural settings. It has been demonstrated that there is a rapid increase in MARV infections in juvenile bats shortly after the waning of maternal immunity, so most juveniles become seropositive for MARV within a few months after birth [14,16].

The lack of MARV transmission by aerosol, an absence of infectious virus in nasal secretions, and the low level of replication of MARV in the lungs of ERBs [19] indicate that a respiratory route is not an important mode of transmission. Orofaecal shedding of MARV was demonstrated in both wild-caught and experimentally infected ERBs [11,17,19,20,22], and the clearance of MARV from the salivary glands of experimentally infected ERBs is slower than from other tissues [18,20,22]. Based on results from experimental studies, it appears that the oral route might be a very efficient or even the main mechanism of MARV transmission among ERBs, with virus shedding peaking after the first week of infection (18, 20). However, it remains questionable if oral bat-to-bat transmission would sustain enzootic circulation of MARV in nature. Testing of oral and rectal swab samples from 815 wild-caught ERB samples during a season of peak MARV transmission in a wild ERB colony in South Africa yielded positive results by qRT-PCR in 11 rectal swabs (1.3%) and 1 oral swab (0.1%). The detection level was significantly higher in faeces than in saliva [15]. Oral and rectal swab samples collected from wild-caught ERBs in Zambia tested negative for MARV [16]. In the only attempt to date of experimental infection of ERBs with MARV via the oronasal route, bats were both virologically and serologically negative up to 21 DPI [17].

It has been hypothesized that bats with bi-annual and synchronous birth pulses would facilitate the persistence of filoviruses in nature [131] and they coincide with periods of increased risk for human infection with marburgviruses [11]. However, gradual loss of passive immunity [21,132] and emergence of a new naïve bat population would create conducive conditions not only for the transmission of a filovirus but also of any other bat-borne pathogens. The results of an ecological study in South Africa suggested that a single, relatively long birthing season, complemented by asynchronous births and potential migration of bats, might contribute to sustained annual MARV circulation in this particular area [15]. Because *M. schreibersii* bats have only one breeding season per year and do hibernate, it would suggest that there are other driving factors involved in Lloviu virus circulation in Europe, such as reactivation of latent infection [32]. In the context of the opposing results and views, more studies would be required to address whether filovirus infection peaks in bats with both single and bi-annual birth pulses are actually a result of fluctuations in bat population susceptibility to infection or rather due to seasonally increased exposure to another virus source. Schuh et al. [132] demonstrated that diminished MARV IgG antibody levels in ERBs following primary infection are not associated with the loss of long-term protective immunity against MARV reinfection, replication, and shedding; thus, a combination of other factors, e.g., seasonal variations in host population, metapopulation dynamics and environmental factors, might contribute to MARV maintenance in nature.

Bats that had been inoculated with MARV by a combination of intraperitoneal and subcutaneous routes seroconverted and developed a sufficient viremia to allow detection by isolation of MARV from different organs, with the highest virus concentration detected in the female reproductive tract (up to 10^7.1^ TCID_50_/g tissue). MARV was also isolated in the mammary gland tissues (10^4.7^TCID_50_/g tissue) collected from a single female 9 DPI [17], and in a subsequent study MARV RNA was detected in vaginal swabs 14 and 21 DPI [19]. The EBOV-specific tropism to female reproductive tissues, particularly in the placenta, where the highest level of replication occurred, and the ability to infect the fetus were recently demonstrated in *M. condylurus* females. These findings demonstrate that vertical transmission might play a role in the maintenance of EBOV in these bats [32].

Increasing reports of the isolation and detection of diverse viruses in bat-associated hematophagous ectoparasites indicate that ectoparasites might at least function as intermediate hosts if not the main players in the maintenance of bat-borne viruses [56,57,133,134,135,136,137]. However, knowledge and robust evidence of their contribution in transmitting viruses, including spreading filoviruses among cave- and forest-dwelling chiroptera, are very limited. Only a single experimental study to evaluate the potential vectorial role of bat-associated ectoparasites in filovirus ecology has been published to date [55]. Consequently, more experimentation is required and other avenues need to be explored to understand the roles that ERBs could play in the ecology of MARV. SC inoculation of ERBs with MARV can be regarded as a surrogate for mechanical transmission, e.g., through skin injury as a result of bites, and collisions or scratches, including biting among ERBs during fighting for dominance in a social structure or biting by hematophagous arthropods.

The combination of the development of viremia in MARV-infected ERBs, the host-specificity of *T. breviceps,* and the species’ regular blood-feeds together make a robust case for a feral transmission cycle for MARV. However, for this to be recognized, a number of steps for a productive filovirus infection need to occur in a vector, including initiation of infection in the midgut, dissemination from the midgut to the hemolymph and secondary tissues, infection of the salivary glands (and sometimes reproductive tissues for vertical transmission to offspring to be possible), and finally release of the virus into saliva, producing a source of horizontal transmission to a receptive vertebrate host [138].

In this study, bat-associated fleas ingested MARV upon feeding on viremic bats and the virus was successfully delivered via IC inoculation. However, delivery of MARV by these two routes did not result in demonstrable infection of the fleas. In fleas fed on viremic animals, viral detection levels were consistent with those demonstrable in the blood of subcutaneously infected animals, and virus concentration in intracoelomically inoculated fleas was lower than that administered in the inoculum, except for day 0 of IC inoculation. These findings indicate that fleas do not support disseminated MARV replication and are refractory to MARV infection, as also evidenced by the absence of demonstrable transmission to naïve bats. Neither seroconversion nor viremia was recorded in any control bat kept in close contact with MARV-infected donors infested with fleas for up to 40 days post-exposure, and all bats remained virologically and serologically negative up to 38 days post-infestation with inoculated fleas. Although our results indicate that *T. breviceps* lacks vectorial competence for MARV, they ought to be viewed with some consideration. Successful infection of a hematophagous insect by a pathogen, followed by replication and spreading to enable oral transmission during feeding or vertical transmission to offspring, is a complex process driven by a number of interlinked factors, including the vector genotype, the environment, and the viral pathogen [139].

Vector competence is the ability of a vector to support pathogen infection, replication, dissemination, and transmission [139,140]. Vectorial capacity is the combination of vector competence with the biology, ecology, and behaviour of the vector, including amongst others, host preference, daily biting rates and survivorship, the length of extrinsic incubation period (the period of time elapsed from a flea becoming infected until it is capable of transmitting the pathogen), and the length of time a vector remains infectious [140,141]. One of the important factors affecting vectorial capacity is the environmental temperature, which not only impacts vector developmental stages but also affects extrinsic incubation and virogenesis [142,143,144]. A number of genetic factors intrinsic to the vector, including infection and escape barriers, need to be overcome before a pathogen is eventually successfully passed on to a susceptible host. Genetic factors influencing vector competence vary between vector species and only specific arboviruses have evolved to conquer these barriers successfully [144].

The mean temperatures during housing of bats and fleas under BSL4 environmental conditions were within the range recorded at Matlapitsi Cave, where we sourced the bats and ectoparasites for this study, and artificial infestation of bats with fleas was within the range recorded under natural settings. Fleas are commonly found on bats and often in considerable numbers. Rodríguez et al. [145] captured 123 (90 females and 33 males) *Hormopsylla fosteri* fleas from a single adult female *Molossops abrasus*. In the study by Fajri and Armiani [71] on the prevalence and intensity of ectoparasites among cave-dwelling bats in Indonesia, up to 11 *T. breviceps* were found per *Rousettus amplexicaudatus,* with a 90% prevalence in the colony, which corresponds to our records.

Flea diversity, diet, abundance, geographic distribution, host association, and biology make them potentially important actors in the etiology and ecology of pathogens. Both adult male and female fleas require regular blood meals, and they easily move between host animals, aided by their ability to jump up to 150 times their body length [60,92,146,147]. The concerning effect of their dietary requirement is that fleas themselves may be hosts to pathogens and thus provide a natural avenue for pathogen dispersal. Fleas are mainly blood vessel feeders. The resultant physical damage to blood vessels creates a probable entry port for pathogens into the blood host. The two commonly known pathways of pathogen transmission by fleas are by the oral route through regurgitation of blood meals when feeding on hosts or by the faecal route by contaminated faecal pellets [92]. The ability of fleas to jump many times their own body length and to move easily through the fur on a bat host might provide a potential mode for intra- and interspecies dispersal of pathogens, as well as of fleas. Taxonomic relatedness between the principal and auxiliary host species may determine what abundance a parasite can achieve on its auxiliary hosts and in turn its ability for cross-species pathogen spread. Krasnov et al. [148] found that the abundance of a flea on its auxiliary hosts decreases with increasing taxonomic distance of these hosts from the principal host. However, in some cases, fleas fed even better on hosts that were phylogenetically distant from their principal host [149]. The ability of certain flea species to feed on phylogenetically distant hosts carries potential epidemiological implications, for example, for humans and animals entering bat roost sites.

The detection of MARV in bat fleas in our study after feeding on viremic bats up to 11 DPI might have important ramifications. For example, consumption of contaminated ectoparasites could provide the mechanism for oral MARV transmission. Although peak viremias in experimentally infected bats tend to be of moderate infectivity and duration, high consumption rates of ectoparasites [150] coupled with high density and roosting behaviour of *Rousettus* bats and regular blood-feeds by ectoparasites might result in increased exposure to the virus. Host grooming appears to be a significant mortality factor for fleas. Greater numbers of fleas are removed by grooming from animals reacting to flea allergens compared with unaffected animals [151]. The veterinary impact of fleas on bats is unknown, but sensitivity to flea bites and allergic dermatitis have been reported in companion animals, while severe puritanism and inflammation have been recorded in humans [92,152].

Many variables that drive vector competence and vector capacity could not be addressed in our study, but the results obtained seem to strongly suggest that bat fleas do not support MARV replication, either following natural ingestion of infectious viremic blood or following artificial inoculation. The negative results of our experimental infection study are supported by the failure to detect MARV in fleas collected from wild-caught bats at Matlapitsi Cave, where enzootic circulation of MARV has been demonstrated.

Overall, this study has revealed that bat fleas lack vectorial capacity to transmit MARV biologically; however, their role in mechanical transmission should not be discounted. Multiple blood-feeds by fleas, intra- and interhost mobility, direct feeding on blood vessels resulting in venous damage, and the roosting behaviour of ERBs provide a potential physical bridge for MARV dissemination amongst densely packed cave-dwelling bats. The virus transfer might take place through inoculation of broken skin, mucosal membranes, and open wounds when fleas engorged with infectious host blood are squashed during auto- and allogrooming, eating, biting, fighting, or via contaminated mouthparts during feeding.

Knowledge and understanding of the mechanisms that drive filovirus maintenance in nature are essential for developing informed risk-prevention strategies and adequate public health education. However, sound evidence for bats as reservoir hosts for orthoebolaviruses is lacking in most African virus studies [153], and there also are numerous challenges in filovirus ecology research, including the biodiversity of the African tropical ecosystem.

Our study contributes to addressing some of the many gaps in our understanding of the complex ecology of filoviruses and responds to the need for a systematic, taxonomically comprehensive One Health approach to investigate natural reservoirs and associated maintenance and cross-species transmission mechanisms and to help focus direction and the scope of future research and surveillance programs.

## Figures and Tables

**Table 1:** Body measurement of *T. breviceps* collected from Egyptian rousette bats at Matlapitsi Cave, South Africa.

Sex/No. Tested	Measurements (µm)			
		Total Length	Abdomen Length	Abdomen Width
F/15	Mean	849.13	474.24	411.88
	SD	130.75	114.92	41.37
	SE	33.76	29.67	10.68
	Range	879.13–1162.28	349.59–755.62	361.33–477.72
M/26	Mean	800.21	445.06	355.29
	SD	65.31	104.59	23.85
	SE	12.81	20.51	4.68
	Range	645.25–937.64	329.62–893.0	318.57–404.49

**Table 2:** Infestation rate of Egyptian rousette bats with *T. breviceps* at Matlapitsi Cave estimated for collections made in October–November 2016, South Africa.

Bat/Flea Count	No. Bats Captured	No. Females	No. Males
Total no. bats captured	109	85	24
Total no. fleas	478	376	102
Infestation rate (%)	97.2	97.6	95.8
Mean ± SD ^a^ (no. fleas/bat)	4.46 ± 3.52	4.47 ± 3.63	4.43 ± 3.27
Range (no. fleas/bat)	0–20	0–20	0–14

^a^ Standard deviation.

## Data Availability

The data presented in this study are available within the article.
